# The Importance of Local and Global Social Ties for the Mental Health and Well-Being of Recently Resettled Refugee-Background Women in Australia

**DOI:** 10.3390/ijerph191710917

**Published:** 2022-09-01

**Authors:** Kate E. Murray, Caroline Lenette, Mark Brough, Katherine Reid, Ignacio Correa-Velez, Lyn Vromans, Robert D. Schweitzer

**Affiliations:** 1 School of Psychology and Counselling, Queensland University of Technology, Brisbane 4000, Australia; 2 School of Social Sciences, Big Anxiety Research Centre, University of New South Wales, Sydney 2052, Australia; 3 School of Public Health and Social Work, Queensland University of Technology, Brisbane 4000, Australia; 4 School of Allied Health, Australian Catholic University, Brisbane 4000, Australia

**Keywords:** social ties, social support, forced displacement, resettlement, Australia, mixed methods research

## Abstract

Social connections are foundational to the human condition and are inherently disrupted when people are forcibly displaced from their home countries. At a time of record high global forced migration, there is value in better understanding how refugee-background individuals engage theirsocial supports or ties in resettlement contexts. A mixed methods research design aimed to understand the complexities of how 104 refugee-background women experienced their social networks in the first few months of resettlement in Australia. One of the research activities involved participants completing a survey with both quantitative and qualitative components. The quantitative analyses identified the impact of post-migration living difficulties that represented social stressors (worry about family, loneliness and boredom, feeling isolated, and racial discrimination) on the women’s mental health outcomes in the months following resettlement. The qualitative data highlighted the complexities of social relationships serving as both stressors and sources of support, and the importance of recognizing extended families and supports around the globe. The findings point to the need for nuanced accounts of the social contexts surrounding refugee resettlement as important influences able to promote trauma-informed and gender sensitive practices to support mental health and well-being in new settings.

## 1. Introduction

Supporting refugee-background individuals, families, and communities to settle in Australia rests within complex sociocultural and political contexts. A trauma-informed approach includes providing a safe environment when responding to the impact of diverse traumatic experiences linked to forced migration for those fleeing persecution, alongside addressing challenging post-migration difficulties associated with housing, language, education, family violence, health, transport, and employment [1,2]. These challenges are magnified for women, especially those who have experienced gender-based violence in situations of political unrest, war, exile, and resettlement [3].

In recognition of this increased risk, some refugee-background women are considered by the United Nations High Commissioner for Refugees (UNHCR) as Women at Risk. This category acknowledges the particularly vulnerable and risky circumstances of women who do not have the protection of a male relative and are in danger of further victimization, harassment, or serious abuse because of their gender [4]. In 2019–2020, the Australian government issued 2345 visas under the Woman at Risk visa (subclass 204) [4]. The visa was introduced in 1989, resulting in more than 25,900 women and their families resettled in Australia under this visa category [4]. This visa category demonstrates concern for the situation of women via an instrumental construction of risk rather than a critical assessment of oppressive power structures surrounding women before, during, and after their forced migration journeys [5,6]. Regardless, the number of refugee-background women resettled in Australia via this visa warrants a specific exploration of their experiences within the first few months of settlement. This mixed methods research focused on the role of the women’s social ties, both globally and locally, and their impact on health and well-being in the early stages of resettlement in Australia through the Women at Risk visa program. The findings informed the development of a trauma-informed needs assessment tool for the sector, that was more sensitive to gender specific concerns and mental health conceptualizations.

### 1.1. Social Ties and Well-Being in the Resettlement Context

Connections to others are a defining part of the human condition and constructing new lives in resettlement contexts involves establishing a new social architecture. These connections must be forged in contexts where settlement service provision is often poorly resourced and where one-size-fits-all policies simply do not work [7,8]. The characteristics of these social arrangements can have significant implications for refugee-background people’s mental health and well-being. For example, there is substantial evidence that social ties both buffer stress and positively influence well-being through direct, moderating, and mediating effects [9]. Further, there are likely gendered differences in how people engage with social networks and influences of broader social networks on an individual’s experiences that should be considered for developing social support interventions [9]. For women in the Women at Risk settlement program, the first few months after arrival represent a time of dynamic shifts within their social networks, and greater understanding of these changes can inform best ways to foster social support.

Social ties are important as means to access social capital (i.e., benefits received by investment in social relationships) [10] including ties developed within one’s cultural group (i.e., bonding social capital) or ties that helps make broader and more diverse inter-group social connections such as across cultural groups (i.e., bridging social capital). These connections are important aspects of the social contexts influencing mental health and well-being in resettlement contexts. Importantly, the extent to which individuals benefit from social ties is gender-based and defined by sociocultural norms that influence who is included and excluded from groups [11].

Initially, refugee-background individuals and families usually (although not always) benefit from intra-group support or bonding social capital [12] before taking on the challenges of bridging social capital beyond their immediate sociocultural community. However, there is an important (albeit less explored) transnational dimension to this process of creating or maintaining connections, which can include loved ones resettled in different countries, living in refugee camps or urban settings in countries of exile, or who remain in their countries of origin. With family connections spread globally, individuals and families must work hard to keep in touch and provide mutual support including via remittances to one another across the world. For example, Losi and Strang [12] showed the urgency of transnational bonding social capital in their study of refugees in Malta, where knowing that family members in other countries were safe was an overriding concern that needed to be addressed early in the settlement process. Bridging social capital is known to be of substantial value in terms of employment, education, and poverty reduction [13,14,15]. Resettlement by nature disrupts social systems, introducing new challenges to re-establish and develop social ties within new contexts. The current research seeks greater clarity on the role and impact of different types of social supports on mental health and well-being for women early in the resettlement context.

### 1.2. A Decolonial Lens

A decolonial approach recognizes the impact of white privilege and how western-based knowledge systems and colonialist principles underpin methodologies and practices, especially in white-majority countries such as Australia. Decolonizing research implies disrupting power dynamics and control that usually sits with (western) researchers and academic institutions, to instead adopt practices that re-value ways of knowing that have been trivialized and ignored because of colonization (e.g., Indigenous or Black knowledge frameworks) [16].

A key challenge to developing social ties that receives limited attention in the refugee studies literature is the broader socio-political context of Australia as a white settler colonial state founded on violence and the ongoing dispossession of Aboriginal and Torres Strait Islander people. The racialized dominant narrative of integration in Australia reflects a colonizer standpoint and the ongoing legacy of imposing white settler values as a deliberate tool to fortify the ‘white nation’ [17]. In this context, the ‘other’, that is, those deemed undesirable and situated outside the white norm, are essentialized and excluded from the ‘white nation’. As McPherson [18] has argued, ‘successful settlement’ in a white settler colonial state effectively means becoming integrated within a dominant white worldview. The expectation of integration reaffirms the white settler identity as dominant and superior [19] and continues to be part of an Indigenous erasure. The lexicon of integration extends colonial practices to debates on migration using new language, but the intent is essentially the same.

For refugee-background people who have already been oppressed through the workings of European and North American colonization in countries of origin, ‘successful settlement’ brings layered and complex meanings due to their ‘salvation’ being founded in a moment of ‘integration’ with the same whiteness that underpinned the historical colonization of their homeland. Moreover, the intersections with gender in regard to the social responsibilities placed on women in families and communities, including within refugee contexts [20] further muddies how we understand social ties. With this background of multi-layered and ultimately messy connections between social ties and well-being, we take inspiration from Tuck [21] who argued for a transformative third space imagination that “more closely matches the experiences of people who, at different points in a single day, reproduce, resist, are complicit in, rage against, celebrate, throw up hands/fists/towels, and withdraw and participate in uneven social structures” (p. 420).

This framing is crucial to consider for both research and service delivery practices in this sector, which are still deeply entrenched in colonialist-imposed interpretations of integration, health, and well-being. Our collective failure to challenge white normativity and privilege as underpinning values in these contexts means that refugee-background individuals, families, and communities rarely encounter practices that acknowledge the nuances and complexities of their lived experiences. That is why we use this lens to outline the contextual specificities of mental health and well-being and the importance of social ties for refugee-background women in Australia and other major resettlement countries such as the United States, United Kingdom, Canada, France, and Sweden [22].

### 1.3. The Current Research

This study draws from a three-year mixed methods study that used several tools including a quantitative survey on social networks with mental health reports alongside qualitative mapping processes with refugee-background women to describe their social networks. These diverse methods aimed to better understand the complexities of how refugee-background women experienced their social networks, including careful consideration of varied social relationships within and between groups, the quality (e.g., support received or discrimination experienced), frequency, forms of interaction (e.g., in-person, via telephone), and the size of those social networks. By using multiple methods and beginning with the participants’ definitions of their “key relationships”, we aimed to generate more complex, nuanced, and multi-faceted descriptions of social ties, and in turn examined their impacts on psychosocial health and well-being. The methods included both commonly used western research methods for this research area (i.e., quantitative survey measures) as well as qualitative methods (i.e., sociogram, digital storytelling, and interview data) that invited new perspectives on social ties. Through data triangulation, we were able to outline similarities and differences across approaches and discuss how social ties were crucial to navigate the challenges and opportunities of a new environment and achieve a sense of well-being. While there was an abundance of data collected for this research, this paper draws from the quantitative and qualitative data focused on the women’s social relationships.

The aims of this study were to describe women’s social networks including the nature and quality of their social relationships, and to assess the associations among dimensions of social networks and participants’ health and well-being in the first months after resettlement in Australia.

## 2. Materials and Methods

Recruitment occurred from 2013 to 2015 in Brisbane, Australia. A total of 104 adult refugee-background women participated. All women were referred to the research through a non-government agency, funded by the Australian Department of Social Services to provide settlement support services to newly arrived refugees. The research was approved by the Queensland University of Technology Human Research Ethics Committee and all women were over age 18, entered Australia in the previous 6 months under the Women at Risk resettlement program, and provided voluntary informed consent to participate. All data presented have been anonymized to protect participants’ identity. We use pseudonyms when referring to participants’ contributions.

Once participants provided informed consent, a research team member and a bicultural health worker provided a survey in the participant’s preferred language, which took approximately 2 h to complete. Surveys were completed in the women’s homes or at the supporting settlement agency. Surveys included both quantitative and qualitative questions. The mixed methods approach leveraged the strengths of both quantitative and qualitative methods and through triangulation of data attempts to address known tensions surrounding the imposition of western (We intentionally use lower case ‘w’ in ‘western’ to decenter colonialist linguistic dominance and discourses) and trauma-focused narratives in research.

The women were on average 32 years old (*SD =* 11.57; range 18–70 years) and had entered Australia within the previous 6 months (*M* = 2.94 months; *SD* = 1.93). Most participating women had entered Australia on Woman-at-Risk (204) visas (84.6%), while the remaining were on either Global Special Humanitarian (202) or Refugee (200) visas. Participants came from diverse countries of origin in Africa (78.8%; including Burundi, Democratic Republic of Congo (DRC), Eritrea, Ethiopia, Kenya, Rwanda, Somalia, South Sudan, and Sudan), South Asia (11.5%; Afghanistan), West Asia (6.7%; including Iran, Iraq, and Syria), and South East Asia (2.9%; including Myanmar and Thailand). Most women self-identified as Christian (60.6%) followed by Muslim (39.4%).

There were two parts to the survey phase of the data collection process. First, the quantitative survey was administered via a structured interview using closed-item measures commonly used and well-established in research with people who have been forcibly displaced (measures in sections a to d below). The researcher worked with the bicultural health worker to ensure these measures were implemented in a standard way. Second, the open-ended qualitative questions were woven into the conversation about the sociogram as outlined further below (in sections e and f).

a. Demographic Data: Questions about the participant, including current age (years), country of birth, months in Australia, relationship status, current household composition, employment status, and educational attainment. Participants stated if they had children and whether they were physically separated from their children. A dichotomous variable was created to indicate whether the women had children (1 = one or more children; 0 = no children).

b. Trauma: Both trauma symptoms and traumatic events were assessed using the Harvard Trauma Questionnaire (HTQ), a widely used and well-established scale [23]. A sum score of the total number of different types of traumatic events experienced or witnessed prior to migration to Australia was calculated from a list of 17 traumatic events (e.g., forced separation from family members, serious injury).

c. Post-migration Living Difficulties: Challenges faced in Australia were assessed using the Post-migration Living Difficulties Checklist (PMLD) developed by Silove and colleagues [24]. An adapted version of the checklist includes 10 items from the original 23-item PMLD Checklist (e.g., difficulties with employment; worry about family not in Australia). Participants indicated whether each problem was *not a problem* (0) to *still a problem today* (4). The measure was recoded to create a comparison between those who viewed the problem as current or serious (serious = 1) versus those who viewed it as not a problem, a small or moderate problem (small = 0), with a sum score generated for the number of serious and current problems women reported (range = 0 to 10).

**Figure 1:**
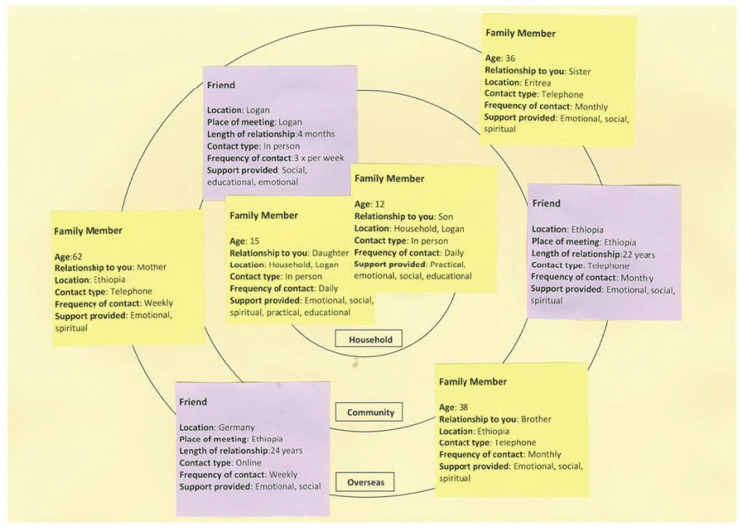
An example sociogram where women identified people within their social network and details about the nature of their relationships.

The types of problems experienced were separated into issues related to emotional factors, which included worry about family, loneliness, and boredom, feeling isolated, and experiences of racial discrimination; and practical problems, which featured communication, transport, employment, immigrant/asylum processes, accessing health and welfare services, and adjusting to cultural life. These two subscales, Emotion-focused PMLD and Practical-focused PMLD, were generated by summing the binary coding of serious versus small problems. Responses range from 0 to 4 for Emotion-focused PMLD and 0 to 6 for Practical-focused PMLD.

d. Mental Health Symptoms: Mental health symptoms were assessed using the 37-item Hopkins Symptom Checklist (HSCL-37), which extends from the HSCL-25 [25] and includes subscales related to participants’ anxiety (10 items; e.g., fearful), depression (15 items; e.g., feel hopeless about the future), and somatic (12 items; e.g., headaches) symptoms. The items were rated on a four-point scale (1 = *not at all* to 4 = *extremely*), with an average score across items taken where higher scores indicate higher levels of distress. The HSCL-37 demonstrated good internal consistency in this research (all α > 0.79) and other research [26].

e. Sociogram: Participants used a visual mapping of social connections exercise to create a sociogram that identified people within their respective networks and summarized details about the nature of their relationships. Yellow post-it notes identified people who were family members, while purple post-it notes identified friends. The post-it notes were categorized according to connections in ‘household’, ‘community’ or ‘overseas’. Figure 1 provides an example of a typical, de-identified sociogram based on a participant’s description of their social arrangements. All sociograms were documented using the same format. This example sociogram has 8 people listed as providing social support.

A sum was generated of the number of key social figures reported: in their social network; by location (household, community, overseas, or unknown); type of support provided (emotional, practical, financial, social, spiritual, educational, or no support); the medium of contact (face to face, phone, Internet, and via applications such as WhatsApp); and frequency of contact (from daily to yearly). Dichotomous variables were generated for each type of support (1 = 1 person or more providing that type of support; 0 = no people providing that type of support) and for whether a person had any social supports in unknown locations (1 = yes, 1 or more social supports in unknown locations; 0 = no social supports in unknown locations). Next, two subscales were generated to indicate whether people received any practical support (a sum of practical, financial, and educational support) and emotional support (a sum of emotional, social, and spiritual support). This resulted in final scores ranging from 0 to 3 for all participants on the two subscales.

f. Open-ended qualitative questions: Interviewers encouraged participants to self-assess or explain their perceptions of their sociogram. For example, “Can you tell me about how these relationships have impacted your resettlement in Australia?”. Participants were encouraged to provide broad responses rather than be constrained by a schedule of questions. The questions were not predetermined but rather were responsive to the conversations that unfolded around the sociogram. This enabled a non-intrusive approach where participants drove the discussions rather than the researcher, as an important alignment with the decolonial lens that guided the research.

g. Digital storytelling: A small group of three participants worked with a facilitator to create digital stories on their resettlement experiences [2]. Using creative narrative techniques and audio-visuals, they shared stories of loss, displacement, and hope, highlighting what contributed to their psychosocial health and well-being through storytelling. This was a trauma-informed approach because it did not rely on a question-answer model to interrogate painful memories. Instead, the women were free to choose the structure and content of their narratives. These videos were not disseminated publicly, as they were created for the women-protagonists. This approach revealed gender-specific narratives that may have been missed using other methods.

In this paper, we focus on the significance of social ties from the sociogram data and the unique insights that mapping participants’ social networks can reveal about well-being. This is done through a concurrent triangulation research design [27] that combines quantitative and qualitative analysis to enable a richer understanding of the experiences of social ties for women resettled in Australia. In this paper, we only report the qualitative analyses drawn from the sociogram conversations and report other qualitative data elsewhere.

### Data Analysis and Interpretation

Using SPSS (IBM, Armonk, NY, USA, version 23) for the quantitative analyses, the data was initially screened to ensure no errors with data entry, address any missing data, and calculate total scores for measures according to scale developer instructions. Demographic or predictor variables (i.e., PMLD and social supports) that had significant bivariate correlations with 1 or more of the mental health outcomes (i.e., trauma symptoms, anxiety symptoms, depression symptoms, and somatic symptoms) were retained for the final regression models. The multivariate assumptions of the final regression models were tested [28]. The plotting of residuals suggests some deviation from a normal distribution of residuals, though this assumption is robust and only extreme deviations are likely to impact findings. We have reported on our quantitative methods and findings elsewhere in further detail [1,29,30].

We used a thematic approach to initiate our qualitative analyses. Drawing on the work of Braun and Clarke [31], we familiarized ourselves with participants’ responses then coded the data to generate initial themes. We then reviewed, grouped, defined, and named themes by considering our research aims. This generated an initial analysis which we shared with one another and reflected on as we considered the full corpus of our mixed method data, and our individual reflexive positioning in response to the data. We drew on Mill’s [32] distinction between analysis and interpretation where interpretation requires meaning making rather than just systematic analysis. Thus, we shared our reflections and understood our task of interpretation as one of building a cohesive argument based on the data. Such an approach favors a more nuanced outline of findings.

## 3. Results

Only 21% of participants reported they were married or in a de facto relationship, with the remaining women reporting being single (49%), widowed (20%), or divorced/separated (10%). Most reported limited English ability (62% = no skills/great difficulty), and most reported having one or more children (61%; *M* = 2.2, *SD* = 2.5). Thus, the majority of the sample reported being single women, with children, and with limited English ability.

The frequencies and characteristics of social connections mapped in sociograms suggest that there were clear patterns of how participating women identified sources of social support (see Table 1). The social ties included a mix of family members and friends in Australia and overseas. In both contexts, participating women had regular contact with many people either face to face (local) or via telephone (local and overseas) daily to twice or more weekly. Across the sociograms, it is at the household and transnational levels that support was most significant. Local, community level social ties were least important, reflecting no doubt the limited time in settlement in Australia and the often-complex community dynamics that can exclude rather than include [11]. We extend our qualitative analysis of the sociogram data in the next section.

### 3.1. Social Networks, Post-Migration Living Difficulties (PMLD), and Mental Health

In the quantitative analyses of relationships between social networks, PMLD and mental health, participating women reported high levels of mental health symptoms (see [29] for a more detailed analysis of mental health symptoms in the first 6 months following settlement). Table 2 provides a correlational analysis of the relationships between social network characteristics and PMLD with the mental health symptoms reported. Across all variables, the correlations between PMLD with all mental health outcomes were significant and consistently the largest. This appears to be driven by the difficulties categorized as emotional challenges, which included worry about family, loneliness and boredom, feeling isolated, and racial discrimination, all of which are linked to elements of social connectivity. Having children was also associated with higher mental health symptoms, thus adding to our understanding of the complexity of children serving as both a crucial support and challenge for the women in early resettlement.

Next, we examined the relative influence of a range of predictive factors within four hierarchical regression models predicting each of the four mental health outcomes: trauma symptoms measured by the HTQ, and symptoms of anxiety, depression, and somatic complaints reported on the HSCL−36. A uniform set of predictor variables were run based on the bivariate correlations, with the first step including pre-migration and more stable characteristics (having children, pre-migration trauma experiences, and having a support person in an unknown location) and the second step including post-migration factors (Emotional and Practical PMLD and emotional social support). Model statistics are provided in Table 3, Table 4, Table 5 and Table 6. All models were significant and accounted for between 18 and 40% of the variance in mental health symptoms.

While there was some variability in significant predictors across the four models, Emotional PMLD was a significant predictor across each model and the most powerful predictor in all models, except when predicting trauma symptoms. In the model predicting trauma symptoms, pre-migration trauma was the strongest predictor (β = 0.35), closely followed by Emotional PMLD (β = 0.26). Emotional PMLD was the only significant predictor in the final model for both anxiety and somatic symptoms. This consistency highlights the pervasive role of social connections across women’s reports of their mental health symptoms and the importance of worry about family, loneliness and boredom, feeling isolated, and racial discrimination in the early stages of resettlement.

While having one or more children had a significant bivariate correlation with all mental health outcomes, it did not remain significant in any of the multivariable models. While significant in all models in Step 1, after entering post-migration factors it was no longer significant. Thus, having children may introduce other issues, such as practical PMLD or general worry about family members, which have a more direct influence on mental health symptoms, though this requires further exploration in future studies.

Across all models, having emotional social support was a protective factor. In all four models, greater levels of emotional social support were associated with lower mental health symptoms, though it was only significant in the model predicting depressive symptoms (β = −0.19, *p* = 0.04).

Unlike in the other three models, there were a range of significant predictors of increased trauma symptoms, including experiencing higher levels of pre-migration trauma events, having social supports in unknown locations, and experiencing greater emotional and practical PMLD. This model also predicted the greatest amount of variance (40%) of total mental health symptoms. Table 6 provides the final regression model output in predicting trauma symptoms.

### 3.2. Qualitative Descriptions of Social Networks

The quantitative analyses take on further significance when social support is examined qualitatively by interpreting the words the women used to describe their sociogram data. Drawing from the women’s commentaries and our team discussions, we identified three common features across participants’ descriptions on the meanings and importance of (i) social support, (ii) household ties and (iii) global ties:

i. The importance of social support to the women was quite clear as they all described the immense significance of their local and overseas connections, often with a deep sense of gratitude.
ii. Children living in the household made up very significant components of their social networks and the significance of attachment to children stood out in the women’s commentaries about their social worlds. This highlights the importance of local ties, which in this context, referred mainly to household ties.
iii. Family and friends overseas were very significant members of their social networks and were no less important because of geographical distance and international borders. For example, in Figure 1, 5 of the 8 members of the participant’s network were in Ethiopia, Eritrea or Germany. Participants highlighted the importance of those networks for social support, but also the strains they can introduce such as added financial pressure or, as in the quantitative data, when the location of family members is unknown. Such transnational connections suggest the importance of global ties, in both positive and challenging ways.

These qualitative descriptions not only show the intrinsic value of social ties but also the difficulty in applying neat binaries (e.g., enablers/obstructors, present/lacking) to the value of social connections. Some contradictions are evident. For example, in the quantitative analyses, the presence of children was positively associated with poorer mental health outcomes, but in the qualitative analyses, children were described as foundational to a sense of meaning, purpose, and enhanced well-being. By making comparisons within and across the different datasets, we gained a more nuanced understanding of the role of social supports for women early in resettlement.

#### 3.2.1. Social Support

The women attached significant importance to their social networks, especially in Australia. They explained the value of ‘not being alone’ as well as the practical value of the help they could derive from supportive friends and family.

Rani spoke of the practical importance of being able to share problems with others through regular connections with local community members:

*Then you cannot be alone because if you face some problems, you can think this is a new problem. Then you go to call someone and you find that they know this problem and they can provide you with the answer and show that is the life in Australia because they have experienced that problem before and they share how they solved that problem. They can show you information that you don’t know, like how you can start a Certificate 3, how you can look for a job and then you can discuss things together. On the holidays and weekends, you can plan to go out and have a BBQ. At the same time, you can discuss how to live in Australia, about how to love the country and how you can resettle. They can discuss anything with you, study, job, responsibilities, shopping, driving, and helping each other financially with bills, if someone has a problem with $100 or $200 you can help*.

Rani, and other respondents, highlighted how social ties provided bridging capital that was crucial for addressing practical PMLD early in resettlement, such as securing work and managing tasks of daily living.

The challenges of settlement in a new country unsurprisingly led to many women worrying about isolation and loneliness, elements highlighted by emotional PMLD in the quantitative component. When discussing their sociograms, the women appreciated that social connections could counter social isolation. As Lina described, social ties present in Australia were very much linked to the global context:

*I can say that when I think about these [local] relationships, I feel like I’m not alone and think that they are my family because everything they do for me is alright. Sometimes I say that these relationships have taken my family’s place and do the things which my family has done for me and they take that place and do everything for me. Like one of the family members, or one of their kids or relatives, so I’m very happy because before I was thinking that before I move and leave my country and go to another country maybe I’ll be like someone who doesn’t have family members or relatives but I’m ok with all of these people*.

Lina described the need to establish interpersonal connections as vital to her happiness, which closely aligns with the quantitative findings. Across both methodologies, the need for support that addresses both practical and emotional PMLD was identified as important for well-being in the early stages of resettlement.

#### 3.2.2. Household Ties

A household predominantly involved participating women as sole parents with young children. In contrast to the ‘risk’ logic of their visa category, the women spoke extensively about their children as the central social meaning and resource for their lives in Australia. When asked about the people most important in their social networks, women with children invariably spoke about their children:

*My only life is my kids. I have no one else*.

(Amina)

*My children are my life*.

(Khadija)

While the care of children was consistently a major day-to-day responsibility for the women, they spoke of their children as supports in settlement. As Zeinab explained:

*Everything I do is for my children; the children make me feel free. My children have been the ones who have impacted the resettlement period. When I’m feeling upset, the girls sing and dance and make me feel happy again*.

Amal spoke of different roles her children took on and how they helped:

*My oldest son provides guidance to our family and helps with talking, housework and helping to organize the family. My sons help me if I’m feeling tired or sick. My youngest son provides me with emotional support*.

As children often learned English quicker, many women relied on their children to translate for them and navigate their way around new neighborhoods. As Rayah describes, practical support from children could range from doing chores around the house to helping with her own ‘homework’ or budgeting and shopping:

*Take me shopping, show me where to buy affordable items. Children help to speak English and will help with my homework and chores around the house. Also, with budgeting*.

Sara describes the role of her children in accessing health care and to communicate with Centrelink, the Australian Government Department responsible for income support:

*My children help me with taking public transport (they learned fast) and to take me to hospital. They also help me with translating (e.g., over the phone) and how to use the internet to report to Centrelink*.

While child-rearing as sole parents might be predominantly seen as a stressor, and has some support from the quantitative findings, the women’s responses suggest that children were an important source of support in the local context. The quantitative measures failed to capture how children served as important sources of support and meaning, which is an important research gap.

#### 3.2.3. Global Ties

The transnational context of social ties was evident in our interpretation of the women’s commentaries both in terms of family and friends. For examples, Aisha said:

*Friend overseas gives encouragement and says “if you are happy in Australia, I’m happy for you”. The relationship with my friend in Cairo gives me hope that she too can come to Australia. When I’m feeling lonely I can call this friend*.

Participants found it helpful to share their troubles with friends going through their own resettlement experiences in another country:

*My friend in England helps by talking about her experiences as a refugee and raising her sons. We talk about our issues and hopes for the future*.

(Amal)

Despite being in different current environments, bonding capital and the emotional support provided by long-standing close relationships were important. These family roles and connections endured despite distance and included advice about challenging topics such as obligations in terms of overseas remittances:

*My mum is living in Sweden now. My mum tells me I do not need to worry about things now because now I’m living in Australia with my family. She tells me I have a new life in Australia and not to send money to others*.

(Sadiya)

Advice from family overseas spoke to their knowledge of the racial complexities of settlement in white Australia and in other white-majority resettlement nations, as well as family and community accountabilities globally:

*They tell you to be punctual in white people’s countries, to avoid just mixing with African culture and to catch up with whites, go inside and try, and you find that you know that’s life, you are part of this community. They tell you to love your country, to do your best and be in the same position as the people who possess the country you’re in. They say that I’m very lucky, it’s good, it’s a blessing, it’s everything, they like it! We did not think we would ever come here because it’s impossible. People overseas are in a bad situation and think that you’re in a good situation and ask you for money. But you don’t have money, you are suffering, you don’t have a job*.

(Rani)

Conversations with family and friends around the globe served as a basis for both positive and negative comparisons to one’s new environment.

## 4. Discussion

Our findings point to the need for nuanced accounts of the social contexts surrounding refugee resettlement and mental health and well-being in new settings. Quantitative data found consistent themes in the importance of worry about family not in Australia, loneliness and boredom, feeling isolated, and racial discrimination in the months following resettlement. These Emotional PMLD challenges were consistently the strongest predictors of mental health symptoms, except for PTSD symptoms where it was the second strongest predictor following pre-migration trauma. These challenges underscore the influence of social ties, both globally and locally, on wellbeing early in the resettlement process. Concurrently, qualitative data highlighted the complexities of social relationships serving as both stressors and sources of support, and the importance of recognizing broad, extended families and supports around the globe. These findings provide important insights on how trauma-informed and gender sensitive practices might better support achieving mental health and well-being in resettlement contexts.

### 4.1. Social Ties as Both Sources of Support and Stress

We found that social ties were both a source of opportunities and resources as well as responsibilities and anxieties. Social ties are fundamental in both experiences of joy and stress, and of connection and isolation and were represented by complex networks that included conversations and advice from the other side of the world, through to the practical support of a multilingual child in helping to navigate an unfamiliar city. Further, there are dynamic and shifting networks of ties in resettlement, not only at the micro level of the individual or the family, but also at a broader transnational level involving social histories of the ‘here’ and ‘there’.

This mirrors the diverse findings we have reported previously on the settlement experiences of the 104 women included in this study [1,2,29,30,33]. Across multiple angles and depending on the analytic categories adopted and methods used, we gained very different insights regarding the strengths and vulnerabilities of this group of women. When we focused on mental health, we found significant experiences of loss [30], concerns about disconnection and loneliness [33] and high levels of psychiatric distress [29]. However, when we examined aspects such as quality of life and protective factors, we found that the vast majority reported having a good quality of life and were satisfied with their health [1].

We argue that the range of findings is not a matter of discordance, rather it reflects the multiple realities of complex lives in which neat binaries obscure rather than assist. The research record often shows well-being and suffering co-existing side by side in people’s realities (for example, [34]), which disrupts the dominant focus on recovery in mental health practice [20]. This ethos is well expressed in Papadopoulos’ [35] notion of adversity activated development recognizing the complexities of human responses to challenging circumstances.

### 4.2. Nuanced Understandings of Bonding and Bridging Capital

Our findings show that social support is critical in both assisting individuals to deal with the impacts of past traumas and losses and in understanding how refugee-background women mitigate the impact of current conditions (PMLD). The objective numbers of people within their social networks and the specific locations of those individuals were not consistently related to mental health symptoms. Rather, the absence of social support as measured by the Emotional PMLD subscale was the most consistent predictor of poor mental health. Reporting greater levels of worry and concern for others and feelings of isolation and alienation were far more important for understanding the women’s distress and spoke to the salience of absent local and transnational social connections as captured by those items. These findings show similar trends to studies of refugee social networks elsewhere in the world [12,14,36,37]. It is not surprising to find in the sociograms that bonding social capital is the core social capital available for women early in settlement.

This points to the need for a more nuanced assessment of bonding social capital. It is clear from our quantitative and qualitative data that, even in simple visual representations of small networks made up exclusively of bonding social capital, there are significant gradients of support at work. More importantly, the flows of support are multidirectional. At the local household level, while the care of children is a significant source of stress and isolation, children are also substantial sources of support. The women’s qualitative data resoundingly spoke of the sense of meaning as mothers and support provided by children in resettlement. In the quantitative data we see that having children is related to higher mental health symptoms in early resettlement, but within more complex regression models post-migration emotional connections more broadly took precedence. Together, the findings highlight both the centrality and complexity of the women’s social networks.

Likewise, at the transnational level, dispersed global diasporas were both sources of concern and support within the qualitative data. Participants commented on the support they received from family and friends overseas but also stressors, such as the pressure experienced to send money to family overseas, which similarly has been found elsewhere [38]. In the quantitative data this was further supported where having a family member in an unknown location significantly predicted trauma symptoms and aligns with other research on the salience of transnational ties for migrants [12,38,39]. In their study of Latin American immigrant women in the United States, Dominguez and Lubitow [38] noted the important supports and potential downsides to transnational social capital, but overall, the benefits appeared to outweigh costs.

Our findings echo concerns from previous studies about the lack of bridging capital among newly arrived refugee communities [11,40,41]. Given the value of bridging capital in terms of settlement and addressing postmigration difficulties—not just for their own sake, but because they can be as much a source of mental stress for refugee communities as the human rights violations that led to forced migration. The results point to the need to better articulate and expand evidence towards practices that build social architecture for refugee-background individuals, families, and communities, both locally and transnationally. Mental health inquiry is dominated by individualized practices of counseling and psychiatric interventions, with questions lingering as to why more systemic interventions are often ignored [42]. The evidence here demonstrates the need for genuine concern for building wider local social connections and supports. While intrinsically, the value of social connections for refugee-background individuals, families and communities is widely appreciated, there is surprisingly sparse evidence to support the need for policies and programs with an explicit focus on supporting bridging capital.

Beyond these more obvious implications from the data we discussed in this paper, our ongoing engagement with this research project over several years and from multiple disciplinary perspectives provides an excellent opportunity to comment on the broader implications of the study. We do this to further contextualize the importance of our findings and their relevance across disciplines. The first implication concerns the decolonial framing we explored in the Introduction, and the second involves a related critique of our mixed-methods methodology.

### 4.3. Decolonial Framing

Refugee-background individuals, families, and communities resettled to countries with a history of colonization and white settler colonial states have to contend with western-imposed definitions of concepts such as family, usually limited to a nuclear family and based on blood relations, and such definitions shape policies concerning family reunification. This can clash with the realities of many who have migrated from countries where the notion of family includes a larger group of significant others, bound by ancestry, ethnicity, shared experiences, and community connections, including scattered transnational networks and diasporas. As our results suggest, geographical distance and international borders did little to dilute the bonds that connected transnational family members as a significant source of support for participants. Despite the responsibilities also attached to these global ties, it was impossible for the women to discuss social supports without including transnational ties.

Entrenched western underpinnings of research and service delivery practices can lead to only partial understandings of complex circumstances impacting on refugee-background individuals’ psychosocial health and well-being. This is concerning because recent evidence highlights that these are ongoing gaps in the sector. For example, Dew et al.’s exploration of the service use experiences of family members of people with disability from Iraqi and Syrian refugee backgrounds [43] indicates that service delivery models fall short of using a decolonial lens. Consequently, there is little progress made in improving the efficacy and cultural safety of such practices.

The current research highlights that a western-imposed conceptualization of family—and the role of women within it—although not static, can overlook the complexities of social networks, including the crucial role of transnational family ties as sources of support to achieve mental health and well-being in resettlement contexts. The qualitative data highlight this as a likely gap in quantitative assessment, calls for a decolonial understanding of sociological concepts such as ‘family’ that goes beyond western definitions [44], and highlights rather than ignores the multiplicities of women’s identities. Such western concepts are readily imposed in research with individuals, families, and communities who have different explanatory frameworks for such notions, without due consideration to sociocultural and geopolitical relevance [32].

Research with refugee-background participants that is trauma-informed should appreciate the wide array of experiences characterized by the term ‘trauma’ and the current complexities surrounding the use of the term ‘trauma-informed’ [35]. While the qualitative data analyzed here were specific to the sociogram data, we argue that broader understandings grounded in participants’ cultural frames of reference rather than western-imposed definitions are important for reflecting the complexities of participants’ identities and experiences. Critical reflection on frames of reference and measurement tools are only one component of much needed decolonization of research practices. Research that more carefully integrates culturally safe and appropriate practices throughout the research process, from conception and assessment to interpretation and dissemination of findings, is needed. The sociogram tool and conversations were easy to administer and anecdotally more engaging for both participants and research staff to create a more expansive engagement that identified areas not captured by the quantitative survey.

### 4.4. Methodological Critique

In Section 4.1, we highlighted how using multiple methods yielded findings from different angles, each approach enriching our understanding of the women’s psychosocial health and well-being. We used quantitative methods (questionnaires) that were ‘validated’ for application in cross-cultural research. Such measures are insufficient to constitute a decolonial research approach because the principles underpinning the design of such established tools are still grounded in western and often biomedical traditions [45]. This is an aspect that requires further consideration, so that cultural safety in research does not become a tokenistic notion. Our choice of qualitative methods (interviews, sociograms, digital storytelling) aimed to highlight the women’s strengths and cultural knowledge to complement quantitative findings.

Initially, our collaborative writing had distinct emphases on a quantitative *or* qualitative aspect, but over time, our scholarship reflected a more integrated approach to reporting findings from a mixed-methods study, as we have done here. There is an abundance of literature on the challenges and criticisms of mixed-methods designs [46,47], so we were aware of the need to pay specific attention to how we integrated quantitative and qualitative analytical lenses to our findings. All authors were experienced in mixed-methods research and understood its value despite the potential difficulties of ‘speaking’ across methodological traditions. We can say with confidence that we have learned from each other, not just in terms of our collaborative writing, but also because of the opportunity to point out the interesting and rich findings that one methodological approach alone might not have uncovered. Equally important, the findings were put to use through the development of a trauma-informed assessment tool for service providers to better capture the complexities of the women’s experiences identified within the research and to move beyond observation to restructuring of existing systems.

## 5. Conclusions

The complex social network of women in the early stages of resettlement represents a frequently overlooked yet foundational element of forcibly displaced women’s well-being. This paper outlined a decolonial lens for this work that used multiple methodologies and aimed to solicit varied perspectives and complexities of social relationships following forced displacement and resettlement. Social ties had a strong influence on the women’s mental health and well-being in the early months following resettlement, including the positive and negative influences of both local and global ties. We advocate for the development of new practices and research paradigms that privilege different ways of knowing such as non-western conceptualizations of family and the need to recognize the complexities inherent to all social relationships. Further development of research and services practices that are trauma-informed and sensitive to gender specific concerns and mental health conceptualizations are needed.

## Figures and Tables

**Table 1:** Characteristics of the social networks reported by participants (*n* = 104).

Variable	Range	M (SD)
People in the sociogram	1–25	7.49 (4.09)
People who provide		
Emotional support	0–20	5.43 (3.76)
Practical support	0–12	3.13 (2.53)
Financial support	0–11	0.72 (1.69)
Social support	0–20	3.22 (3.96)
Spiritual support	0–20	2.23 (3.55)
Educational support	0–20	1.67 (3.41)
No support	0–7	0.70 (1.25)
Location of support		
Household	0–8	2.93 (2.08)
Community	0–8	1.37 (1.67)
Overseas	0–14	2.88 (2.56)
Unknown	0–4	0.23 (0.63)
Medium of contact		
Face to face	0–12	4.00 (2.79)
Telephone	0–12	2.63 (2.34)
Internet	0–12	0.59 (1.78)
Web-based apps	0–5	0.29 (0.95)
Frequency of contact		
Daily	0–10	3.71 (2.40)
Twice+ Weekly	0–10	1.04 (1.81)
Weekly	0–7	0.84 (1.27)
Fortnightly	0–8	0.70 (1.26)
1–3 times per Month	0–3	0.12 (0.40)
Monthly	0–4	0.35 (0.75)
Less than Monthly	0–5	0.17 (0.68)
Yearly	0–2	0.10 (0.33)
No contact	0–4	0.36 (0.78)

**Table 2:** Correlations between social supports, post-migration living difficulties and trauma events with mental health outcomes among women resettled in Australia (*n* = 104).

	Trauma Symptoms	Anxiety Symptoms	Depression Symptoms	Somatic Symptoms
Number of people in sociogram	−0.033	−0.015	0.085	0.096
Number in household	−0.152	−0.066	−0.080	0.010
Number in community	−0.096	−0.055	0.012	0.017
Number overseas	0.040	0.011	0.144	0.084
Location unknown (1 = yes; 0 = no)	0.228 *	0.182	0.119	0.197 *
Have children (1 = yes; 0 = no)	0.230 *	0.206 *	0.277 **	0.252 **
Emotional support	−0.090	−0.190	−0.202 **	−0.133
Practical support	−0.127	−0.181	−0.117	−0.044
Total trauma events	0.475 **	0.134	0.275 **	0.268 **
Total PMLD	0.475 **	0.285 **	0.376 **	0.360 **
Practical PMLD	0.314 **	0.153	0.158	0.201 *
Emotional PMLD	0.438 **	0.304 **	0.453 **	0.375 **

Note. * *p* < 0.05, ** *p* < 0.01, PMLD = post-migration living difficulties.

**Table 3:** Hierarchical linear regression predicting trauma symptoms from pre-migration and post-migration factors in a sample of adult women in resettlement.

	B	SE B	95% CI	β	*p*-Value
Step 1					
Constant	1.19	0.12	0.95, 1.44	--	<0.01
Children	0.22	0.11	0.00, 0.43	0.17	0.05
Pre-migration trauma	0.06	0.01	0.04, 0.08	0.44	<0.01
Unknown location	0.32	0.14	0.05, 0.60	0.20	0.02
Step 2					
Constant	1.22	0.17	0.90, 1.55	--	<0.01
Children	0.12	0.11	−0.09, 0.33	0.09	0.27
Pre-migration trauma	0.05	0.01	0.02, 0.07	0.35	<0.01
Unknown location	0.28	0.13	0.02, 0.54	0.17	0.04
Emotional PMLD	0.17	0.06	0.06, 0.29	0.26	<0.01
Practical PMLD	0.10	0.05	0.01, 0.20	0.17	0.04
Emotional support	−0.09	0.05	−0.20, 0.01	−0.14	0.09
Final model: F (6, 97) = 10.97, *p* < 0.001, R^2^ = 0.40					

Note. Children were coded as dichotomous (1 = 1 or more children; 0 = no children); Unknown location was coded as dichotomous (1 = at least one family member in an unknown location; 0 = none in unknown locations); PMLD = post-migration living difficulties.

**Table 4:** Hierarchical linear regression predicting anxiety symptoms from pre-migration and post-migration factors in a sample of adult women in resettlement.

	B	SE B	95% CI	β	*p*-Value
Step 1					
Constant	12.48	1.38	9.74, 15.23	--	<0.01
Children	2.40	1.19	0.05, 4.75	0.20	0.05
Pre-migration trauma	0.12	0.13	−0.13, 0.37	0.09	0.34
Unknown location	2.87	1.56	−0.22, 5.96	0.18	0.07
Step 2					
Constant	13.60	1.91	9.81, 17.39	--	<0.01
Children	1.52	1.21	−0.88, 3.93	0.12	0.21
Pre-migration trauma	0.04	0.13	−0.22, 0.30	0.03	0.76
Unknown location	2.44	1.51	−0.56, 5.44	0.15	0.11
Emotional PMLD	1.66	0.67	0.34, 2.97	0.25	0.01
Practical PMLD	0.45	0.55	−0.65, 1.54	0.08	0.42
Emotional support	−1.14	0.61	−2.36, 0.08	−0.18	0.07
Final Model: F (6, 97) = 3.54, *p* < 0.01, R^2^ = 0.18					

Note. Children were coded as dichotomous (1 = 1 or more children; 0 = no children); Unknown location was coded as dichotomous (1 = at least one family member in an unknown location; 0 = none in unknown locations); PMLD = post-migration living difficulties.

**Table 5:** Hierarchical linear regression predicting depressive symptoms from pre-migration and post-migration factors in a sample of adult women in resettlement.

	B	SE B	95% CI	β	*p*-Value
Step 1					
Constant	17.68	1.79	[14.12, 21.24]	--	<0.01
Children	4.05	1.54	[1.00, 7.09]	0.25	0.01
Pre-migration trauma	0.41	0.16	[0.80, 0.73]	0.23	0.02
Unknown location	2.27	2.02	[−1.73, 6.27]	0.11	0.26
Step 2					
Constant	18.85	2.34	[14.19, 23.50]	--	<0.01
Children	2.95	1.49	[−0.01, 5.90]	0.18	0.05
Pre-migration trauma	0.25	0.16	[−0.08, 0.57]	0.14	0.13
Unknown location	1.29	1.86	[−2.39, 4.98]	0.06	0.49
Emotional PMLD	3.43	0.82	[1.81, 5.04]	0.39	<0.01
Practical PMLD	0.16	0.68	[−1.19, 1.50]	0.02	0.82
Emotional support	−1.61	0.76	[−3.11, −0.12]	−0.19	0.04
Final model: F (6, 97) = 7.31, *p* < 0.001, R^2^ = 0.31					

Note. Children were coded as dichotomous (1 = 1 or more children; 0 = no children); Unknown location was coded as dichotomous (1 = at least one family member in an unknown location; 0 = none in unknown locations); PMLD = post-migration living difficulties.

**Table 6:** Hierarchical linear regression predicting somatic symptoms from pre-migration and post-migration factors in a sample of adult women in resettlement.

	B	SE B	95% CI	β	*p*-Value
Step 1					
Constant	14.97	1.41	12.18, 17.76	--	<0.01
Children	2.90	1.20	0.51, 5.29	0.22	0.02
Pre-migration trauma	0.31	0.13	0.05, 0.56	0.22	0.02
Unknown location	3.12	1.58	−0.02, 6.25	0.18	0.05
Step 2					
Constant	15.30	1.93	11.47, 19.13	--	<0.01
Children	2.13	1.23	−0.30, 4.57	0.17	0.09
Pre-migration trauma	0.19	0.13	−0.07, 0.46	0.14	0.15
Unknown location	2.57	1.53	−0.46, 5.60	0.15	0.10
Emotional PMLD	1.96	0.67	0.63, 3.29	0.28	<0.01
Practical PMLD	0.51	0.56	−0.60, 1.61	0.09	0.36
Emotional support	−0.87	0.62	−2.11, 0.36	−0.13	0.16
Final Model: F (6, 97) = 5.33, *p* < 0.001, R^2^ = 0.25					

Note. Children were coded as dichotomous (1 = 1 or more children; 0 = no children); Unknown location was coded as dichotomous (1 = at least one family member in an unknown location; 0 = none in unknown locations); PMLD = post-migration living difficulties

## Data Availability

The data presented in this study are available on request from the corresponding author. The data are not publicly available due to the personal nature of the data.
